# A comprehensive evaluation of single nucleotide polymorphisms associated with osteosarcoma risk: A protocol for systematic review and network meta-analysis

**DOI:** 10.1097/MD.0000000000020486

**Published:** 2020-06-26

**Authors:** Zhuo-Miao Ye, Ming-Bo Luo, Chi Zhang, Jing-Hui Zheng, Hong-Jun Gao, You-Ming Tang

**Affiliations:** a Ruikang School of Clinical Medicine; b Graduate School, Guangxi University of Chinese Medicine; c Department of Cardiology; d Department of Urinary Surgery, Ruikang Hospital Affiliated to Guangxi University of Chinese Medicine, Nanning, Guangxi; e Department of Gastroenterology, Ruikang Hospital Affiliated to Guangxi University of Chinese Medicine, Nanning, China.

**Keywords:** case-control study, model of inheritance, network meta-analysis, osteosarcoma, susceptibility

## Abstract

**Background:**

Single nucleotide polymorphisms (SNPs) have been inconsistently associated with osteosarcoma (OS) risk. This meta-analysis aimed to synthesize relevant data on SNPs associated with OS.

**Methods:**

Databases were searched to identify association studies of SNPs and OS published through January 2020 from the databases of PubMed, Web of Science, Embase, Cochrane Library, China National Knowledge Infrastructure, the Chinese Science and Technology Periodical Database, and Wan fang databases. Network meta-analysis and Thakkinstian algorithm were used to select the most appropriate genetic model, along with false positive report probability for noteworthy associations. The methodological quality of data was assessed based on the STrengthening the REporting of Genetic Association Studies statement Stata 14.0 will be used for systematic review and meta-analysis.

**Results:**

This study will provide a high-quality evidence to find the SNP most associated with OS susceptibility and the best genetic model.

**Conclusions:**

This study will explore which SNP is most associated with OS susceptibility.

**Registration:**

INPLASY202040023.

## Abstract 2

Supplemental Digital Content is available in the text

## 1 Introduction

Osteosarcoma (OS), which are the most frequently detected bone cancers,is the third most common cancer type in children and in young adults.^[1]^ OS, which are mainly detected in the femur (42%), tibia (19%), humerus (10%), skull and jaw (8%), and pelvis (8%),^[2]^ has different subtypes, and most of them are high grade and aggressive. Metastases occur in about 20% of patients with OS and most metastasize to bone marrow, liver, brain, and other tissues. OS are resistant to some chemotherapy drugs because of their aggressive biological behavior.^[2]^ But we still do not know why it is so aggressive, which has led to the slow development of treatment for OS in recent years.^[3]^ Genetic variations in patients with OS can lead to extensive changes in the response and toxicity of chemotherapy drugs. For example, the variation of DMET gene was significantly correlated with tumor necrosis outcome and survival rate.^[4]^ Single nucleotide polymorphism (SNPs), which are inherited single base changes in exonic or intronic regions. Some SNPs have been found to alter gene expression and function, or be in linkage disequilibrium with causal loci associated with cancer risk and/or prognosis. Numerous studies have found common SNPs within biologically plausible pathways that have been hypothesized to contribute to OS etiology, such as TP53,^[5]^ TCF21,^[6]^ ERCC2,^[7]^ CTLA-4.^[8]^ Most of these studies; however, have limited statistical power to detect small-effect SNPs and the results are often inconsistent and thus inconclusive. Some systematic reviews have evaluated the evidence regarding SNPs in individual genes related to OS,^[9–12]^ but few reviews have comprehensively summarized and evaluated all SNPs related to OS. Our study was to comprehensively evaluate significant SNPs associated with OS susceptibility. There is a lack of evidence to indicate which genetic model is most appropriate to identify associations of SNPs with OS; thus, instead of assuming the underlying genetic model, we applied various approaches to select the most appropriate genetic models of inherence and to measure the reliability of the associations.

## 2 Materials and methods

This study was conducted in accordance with the preferred reporting items for systematic reviews and meta-analyses guidelines and the protocol was registered in the INPLASY database. Ethical approval will not be necessary since this systematic review and meta-analysis will not contain any private information of participants or violate their human rights.

### 2.1 Criteria for the included studies in the review

#### 2.1.1 Types of studies

Case-control study, published in either English or Chinese that concern the susceptibility of the SNPs to the OS, will be incorporated in our review. No limitations of publication status or data will be settled. Studies reported in full-text will be screened for inclusion. Additionally, those registered in the trials registries but have not been published will be contacted to ascertain whether the complete data is available. A study was excluded if it was a repeat report, conference report, thesis, review paper, or animal study, or had insufficient data for genotyping distribution calculation. Studies in which SNPs demonstrated a departure from Hardy–Weinberg equilibrium (HWE) in controls were excluded. A SNP was included only if more than 2 studies meeting the aforementioned criteria evaluated this genetic variant. The references of all eligible studies were manually screened to ensure that all relevant studies were included.

#### 2.1.2 Types of participants

We will include trials of OS patients whose the diagnosis was based on criteria not aforementioned or diagnosis criteria were not clearly defined. Noncancer controls may be healthy or have nonmalignant diseases. Studies were considered only if the studied population was taken serum samples before prior chemoradiotherapy and cancer risk was the outcome. No restrictions were placed on age, gender, country, or tumor stage.

### 2.2 Search strategy

#### 2.2.1 Electronic searches

Studies published through January 2020 that compared frequency differences in SNPs between OS patients and noncancer controls were identified from PubMed, Web of Science, Embase, Cochrane Library, China National Knowledge Infrastructure, the Chinese Science and Technology Periodical Database, and Wanfang databases, with no language limits. The search strategy was based on the following search terms: “single nucleotide polymorphism,” “SNP,” “ osteosarcoma,” and “osteogenic sarcoma.” Details regarding the search terms are available in the Supplementary Materials S1.

### 2.3 Data collection

#### 2.3.1 Selection of studies

Two reviewers (ZY and CZ) conducted the selection process independently, with cases of disagreement resolved by discussion or consulting a third reviewer (JZ).

**Figure 1:**
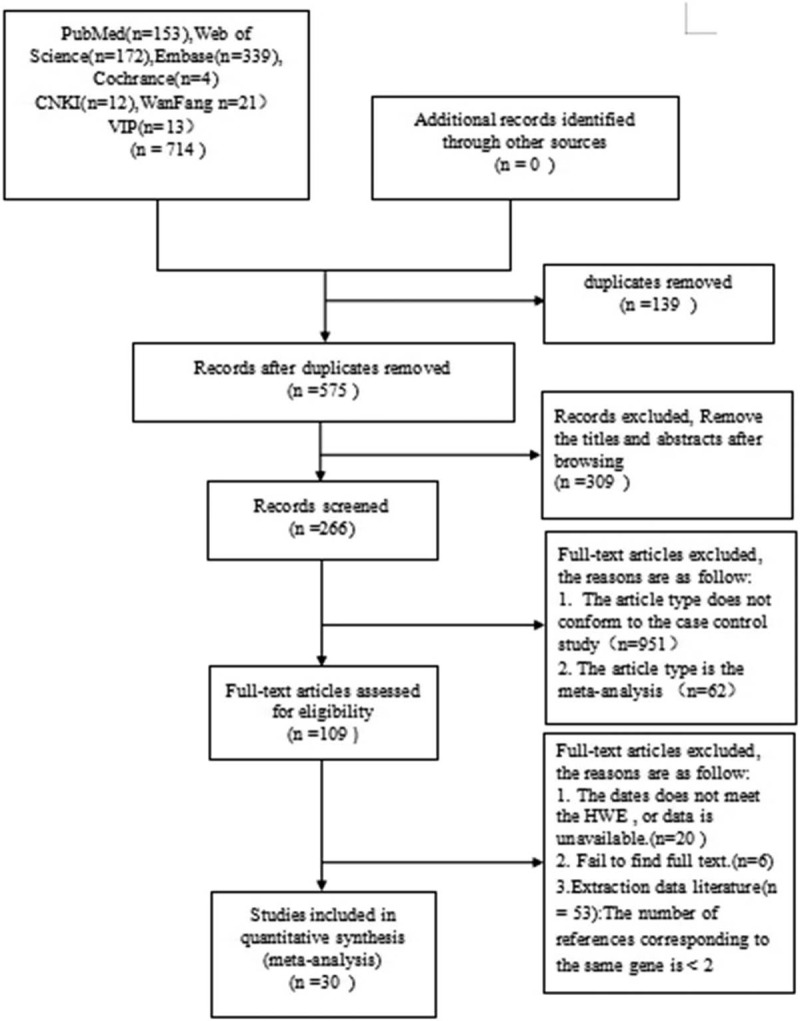
PRISMA flow diagram of literature search and selection. PRISMA = preferred reporting items for systematic review and meta-analysis.

After removing duplicate and irrelevant articles, 2 reviewers will create 2 lists of potential studies which will be checked against each other by a supervisor (JZ) to ascertain a preliminary list. Further identification of eligible articles from the preliminary list will be completed by 2 reviewers through applying the preplanned inclusion/exclusion criteria. A third reviewer (JZ) will make a judgment when disagreements occur. Figure 1 is the preferred reporting items for systematic review and meta-analysis flow diagram illustrating the procedure of study selection.

#### 2.3.2 Data extraction and qualitative evaluation

Data extracted from individual papers include: author, year of publication, country, sample size, type of controls, sex composition, age of diagnosis, and details of target SNPs, including genotyping methods, frequencies of genotypes and derivation from HWE. The methodological quality of data was assessed based on the STrengthening the REporting of Genetic Association Studies statement.^[13]^ The following questions were rated using a 2-point scale (0/no, 1/yes):

(1) Is there description of genotyping methods?
(2) Is there population stratification assessment?
(3) Is there description of methods for inferring genotypes?
(4) Is there description whether HWE holds in controls?
(5) Is there description of whether the study is the first report or a replication effort, or both?
(6) Is information given on eligibility criteria and matching criteria for matched case-control studies?
(7) Is there description of statistical methods and software version used?
(8) Is there description to address and correct for relatedness among subjects? and
(9) Is the data adequate?

Two reviewers conducted the rating independently and a third reviewer was consulted for consensus if disagreement occurred.

### 2.4 Dealing with missing data

If the data of potential studies are missing, insufficient, or vague, we will attempt to contact the corresponding authors to retrieve the necessary data through email or telephone. The studies will be excluded if we cannot obtain the relevant data via the aforementioned approaches.

### 2.5 Statistical analysis

For controls of each study, HWE was estimated using the goodness-of-fit test. For pairwise meta-analysis, a fixed- or random-effects pooled odds ratio (OR) with 95% confidence intervals were calculated, depending on degree of heterogeneity under 6 genetic models (allele contrast model, homozygous model, heterozygous model, dominant model, recessive model, and over-dominant model). Heterogeneity was quantified with the *I*^2^ statistic and *P*-value; a *I*^2^ statistic <50% and a *P* > .1 indicated low heterogeneity between studies, in which case the fixed-effect model was employed, otherwise, random effects model will be used. For significant SNPs with evidence of heterogeneity in meta-analysis, assessment of sources of heterogeneity was employed using subgroup analysis if sufficient data existed. Publication bias was assessed using the Begg and Egger tests.

#### 2.5.1 Network meta-analysis

A random-effects network meta-analysis within a Bayesian framework was conducted using the GeMTC software (v 0.14.3).^[14]^ Four parallel Markov chain Monte Carlo simulations were run for a 20,000-stimulation burn-in phase and an additional 50,000-stimulation phase. Convergence was satisfied with a potential scale reduction factor value of 1.0 as the cut-off value. Consistency, referring to agreement between direct and indirect comparisons in terms of effect estimates, was evaluated by comparing consistency model with inconsistency model in terms of standard deviation of the random effect. The inconsistency model was used when an obvious deviation was detected; otherwise, the consistency model was used. This Bayesian approach was used to rank the probability of each genetic model for risk assessment for OS and corresponding rank probability plots were generated.

#### 2.5.2 False positive report probability (FPRP)

We further compared genetic models to select the most appropriate model using the algorithm by Thakkinstian et al.^[15]^ A SNP consists of a dominant allele (G) and a recessive allele (g). Pairwise differences of GG versus gg (D1), Gg versus gg (D2), and GG versus Gg (D3) were calculated as pooled OR1, OR2, and OR3, respectively, along with 95% confidence intervals, in the pairwise meta-analysis. The most appropriate genetic model was determined to be: recessive model if OR1 = OR3 ≠ 1 and OR2 =1, dominant model if OR1 = OR2 ≠ 1 and OR3 =1, a complete over-dominant model if OR2 = 1/OR3 ≠1 and OR1 = 1, or codominant model if OR1 > OR2 > 1 and OR1 > OR3 > 1 (or if OR1 < OR2 < 1, and OR1 < OR3 < 1). To assess the noteworthiness of the normally significant SNPs under the most appropriate genetic model determined by network meta-analysis or Thakkinstian’ algorithm, FPRP was calculated assuming 3 levels of prior probabilities (low: 0.1; moderate: 0.01; high: 0.001) and an OR of 1.5, as previously described.^[16,17]^ Significant SNPs with a FPRP value <0.2 were considered noteworthy.^[16]^

#### 2.5.3 Diagnostic meta-analysis

Diagnostic meta-analysis was conducted to determine sensitivity and specificity of SNPs in predicting OS risk using the Meta-DiSc software^[18]^ just as Zhang's study did.^[19]^ The correlation between the pooled sensitivity and specificity was estimated using the summary receiver-operating characteristic curve and its area under the curve; positive likelihood ratio, negative likelihood ratio, and diagnostic odds ratio were calculated accordingly. Spearman correlation analysis was used to evaluate heterogeneity related to threshold effect.

#### 2.5.4 Subgroup analysis

We will conduct a subgroup analysis of the SNPs most associated with OS, according to race, type of virus infection, age, sex, and so on.

#### 2.5.5 Sensitivity analysis

Sensitivity analysis will be conducted to check the robustness and reliability of pooled outcome results.

#### 2.5.6 Reporting bias

Publication bias will be assessed with funnel plot and Egger regression analysis if sufficient trials (≥10 trials) are included.

## 3 Discussion

Some systematic reviews have evaluated the evidence regarding SNPs in individual genes related to OS,^[9–12]^ but few reviews have comprehensively summarized and evaluated all SNPs related to OS, and they included a smaller sample. Moreover, risk association analysis based on a priori genetic model may be misleading if an inappropriate genetic model was assumed. Given that, in this study, we applied various approaches to select the most appropriate genetic models of inherence and to measure the reliability of the associations between SNPs and OS. To identify the most appropriate model for OS risk association, both network meta-analysis and Thakkinstian's algorithm were used. Those SNPs we obtained through analysis of our study may assist clinicians in assessing the prognosis of OS patients and selecting appropriate targets therapy.^[20]^ We believe that this systematic review will find the SNP most associated with OS susceptibility and select the most appropriate genetic models.

## Author contributions

**Analysis planning:** Jing-Hui Zheng, Hong-Jun Gao, Chi Zhang.

**Conceptualization:** Jing-Hui Zheng, Hong-Jun Gao, Chi Zhang.

**Data curation:** Zhuo-Miao Ye, Ming-Bo Luo.

**Draft manuscript:** Zhuo-Miao Ye, Ming-Bo Luo.

**Investigation:** Jing-Hui Zheng.

**Manuscript editing:** Zhuo-Miao Ye, Jing-Hui Zheng.

**Methodology:** Zhuo-Miao Ye, Jing-Hui Zheng, Chi Zhang.

## Supplementary Material

Supplemental Digital Content

## References

[1] Gianferante DM Mirabello L Savage SA Germline and somatic genetics of osteosarcoma - connecting aetiology, biology and therapy. J Nat Rev Endocrinol 2017;13:480–91. 10.1038/nrendo.2017.16 28338660

[2] Tsiambas E Fotiades PP Sioka C Novel molecular and metabolic aspects in osteosarcoma. J BUON 2017;22:1595–8. 29332359

[3] Ragland BD Bell WC Lopez RR Cytogenetics and molecular biology of osteosarcoma. Lab Invest 2002;82:365–73. 11950895 10.1038/labinvest.3780431

[4] Bhuvaneshwar K Harris M Gusev Y Genome sequencing analysis of blood cells identifies germline haplotypes strongly associated with drug resistance in osteosarcoma patients. BMC Cancer 2019;19:357. 30991985 10.1186/s12885-019-5474-y PMC6466653

[5] Ru JY Cong Y Kang WB Polymorphisms in TP53 are associated with risk and survival of osteosarcoma in a Chinese population. Int J Clin Exp Pathol 2015;8:3198–203. 26045840 PMC4440149

[6] Jiang Z Zhang W Chen Z Transcription factor 21 (TCF21) rs12190287 polymorphism is associated with osteosarcoma risk and outcomes in East Chinese population. Med Sci Monit 2017;23:3185–91. 28663539 10.12659/MSM.905595 PMC5503230

[7] Wang ZM Wu N Association between XRCC1 and ERCC2 gene polymorphisms and development of osteosarcoma. Int J Clin Exp Pathol 2016;9:223–9.

[8] Qiao G Miao H Yi Y Genetic association between CTLA-4 variations and osteosarcoma risk: case-control study. Int J Clin Exp Pathol 2016;9:9598–602.

[9] Moghimi M Sobhan MR Jarahzadeh MH Association of GSTM1, GSTT1, GSTM3, and GSTP1 genes polymorphisms with susceptibility to osteosarcoma: a case- control study and meta-analysis. Asian Pac J Cancer Prev 2019;20:675–82. 30909663 10.31557/APJCP.2019.20.3.675 PMC6825775

[10] Asnafi AA Behzad MM Ghanavat M Singe nucleotide polymorphisms in osteosarcoma: pathogenic effect and prognostic significance. Exp Mol Pathol 2019;106:63–77. 30528563 10.1016/j.yexmp.2018.12.002

[11] Bilbao-Aldaiturriaga N Patino-Garcia A Martin-Guerrero I Cytotoxic T lymphocyte-associated antigen 4 rs231775 polymorphism and osteosarcoma. Neoplasma 2017;64:299–304. 28052683 10.4149/neo_2017_218

[12] Hu YY Du XY Zhan AL Vascular endothelial growth factor polymorphisms are associated with osteosarcoma susceptibility. Oncotarget 2016;7:47711–9. 27351225 10.18632/oncotarget.10278 PMC5216973

[13] Little J Higgins JP Ioannidis JP Strengthening the reporting of genetic association studies (STREGA): an extension of the STROBE statement. J Hum Genet 2009;125:131–51. 10.1007/s00439-008-0592-7 19184668

[14] van Valkenhoef G Lu G de Brock B Automating network meta-analysis. J Res Synth Methods 2012;3:285–99. 10.1002/jrsm.1054 26053422

[15] Thakkinstian A McElduff P D’Este C A method for meta-analysis of molecular association studies. J Stat Med 2005;24:1291–306. 10.1002/sim.2010 15568190

[16] Wacholder S Chanock S Garcia-Closas M Assessing the probability that a positive report is false: an approach for molecular epidemiology studies. J Natl Cancer Inst 2004;96:434–42. 15026468 10.1093/jnci/djh075 PMC7713993

[17] Lohmueller KE Pearce CL Pike M Meta-analysis of genetic association studies supports a contribution of common variants to susceptibility to common disease. J Nat Genet 2003;33:177–82. 10.1038/ng1071 12524541

[18] Zamora J Abraira V Muriel A Meta-DiSc: a software for meta-analysis of test accuracy data. J BMC Med Res Methodol 2006;6:31. 10.1186/1471-2288-6-31 PMC1552081 16836745

[19] Zhang C Ye Z Zhang Z A comprehensive evaluation of single nucleotide polymorphisms associated with hepatocellular carcinoma risk in Asian populations: a systematic review and network meta-analysis. Gene 2020;735:144365. 31935498 10.1016/j.gene.2020.144365

[20] Zhang C Zheng JH Lin ZH Profiles of immune cell infiltration and immune-related genes in the tumor microenvironment of osteosarcoma. Aging 2020;12:3486–501. 32039832 10.18632/aging.102824 PMC7066877

