# Supplementary material for: A comprehensive evaluation of single nucleotide polymorphisms associated with osteosarcoma risk: A protocol for systematic review and network meta-analysis
Source: Medicine (Baltimore). 2020 Jun 26;99(26):e20486. doi: 10.1097/MD.0000000000020486 (PMC7328971; doi:10.1097/MD.0000000000020486)
Supplement: Supplemental Digital Content [file medi-99-e20486-s001.docx]

**Pubmed**

Search **((((((((((Osteosarcomas[Title/Abstract]) OR Osteosarcoma Tumor[Title/Abstract]) OR Osteosarcoma Tumors[Title/Abstract]) OR Tumor, Osteosarcoma[Title/Abstract]) OR Tumors, Osteosarcoma[Title/Abstract]) OR Sarcoma, Osteogenic[Title/Abstract]) OR Osteogenic Sarcomas[Title/Abstract]) OR Sarcomas, Osteogenic[Title/Abstract]) OR Osteogenic Sarcoma[Title/Abstract])) AND ((((((Nucleotide Polymorphism, Single[Title/Abstract]) OR Nucleotide Polymorphisms, Single[Title/Abstract]) OR Polymorphisms, Single Nucleotide[Title/Abstract]) OR Single Nucleotide Polymorphisms[Title/Abstract]) OR SNPs[Title/Abstract]) OR Single Nucleotide Polymorphism[Title/Abstract])**
